# Efficacy of auxetic lattice structured shoe sole in advancing footwear comfort—From the perspective of plantar pressure and contact area

**DOI:** 10.3389/fpubh.2024.1412518

**Published:** 2024-06-19

**Authors:** Jifa Zhang, Shizhu Lu, Yadie Yang, Yiwen Liu, Yuqing Guo, Hongrui Wang

**Affiliations:** ^1^Department of Industrial Design and Engineering, School of Art and Design, Guangdong University of Technology, Guangzhou, Guangdong, China; ^2^School of Fashion and Textiles, The Hong Kong Polytechnic University, Hong Kong, China; ^3^Department of Digital Media, Software Engineering Institute of Guangzhou, Guangzhou, Guangdong, China

**Keywords:** auxetic lattice structures, perceived comfort, 3D printing footwear, shoe sole, pedobarographic analysis, footwear biomechanics, plantar health

## Abstract

**Introduction:**

Designing footwear for comfort is vital for preventing foot injuries and promoting foot health. This study explores the impact of auxetic structured shoe soles on plantar biomechanics and comfort, motivated by the integration of 3D printing in footwear production and the superior mechanical properties of auxetic designs. The shoe sole designs proposed in this study are based on a three-dimensional re-entrant auxetic lattice structure, orthogonally composed of re-entrant hexagonal honeycombs with internal angles less than 90 degrees. Materials fabricated using this lattice structure exhibit the characteristic of a negative Poisson's ratio, displaying lateral expansion under tension and densification under compression.

**Methods:**

The study conducted a comparative experiment among three different lattice structured (auxetic 60°, auxetic 75° and non-auxetic 90°) thermoplastic polyurethane (TPU) shoe soles and conventional polyurethane (PU) shoe sole through pedobarographic measurements and comfort rating under walking and running conditions. The study obtained peak plantar pressures (PPPs) and contact area across seven plantar regions of each shoe sole and analyzed the correlation between these biomechanical parameters and subjective comfort.

**Results:**

Compared to non-auxetic shoe soles, auxetic structured shoe soles reduced PPPs across various foot regions and increased contact area. The Auxetic 60°, which had the highest comfort ratings, significantly lowered peak pressures and increased contact area compared to PU shoe sole. Correlation analysis showed that peak pressures in specific foot regions (hallux, second metatarsal head, and hindfoot when walking; second metatarsal head, third to fifth metatarsal head, midfoot, and hindfoot when running) were related to comfort. Furthermore, the contact area in all foot regions was significantly associated with comfort, regardless of the motion states.

**Conclusion:**

The pressure-relief performance and conformability of the auxetic lattice structure in the shoe sole contribute to enhancing footwear comfort. The insights provided guide designers in developing footwear focused on foot health and comfort using auxetic structures.

## 1 Introduction

Comfort is paramount in the manufacture of footwear, often subjectively assessed by the wearer. While subjective opinions of wearers can offer valuable insights into the comfort levels of shoes, this feedback is typically limited to descriptive terms, failing to quantify the reasons behind comfort or discomfort. However, footwear designers need to explore the relationship between perceived comfort and physical measurements, such as the contact pressure at the foot-shoe interface, to identify the impact of specific design features on footwear comfort. Many aspects of foot health can be altered by the distribution of plantar pressure and the sensation of comfort, with numerous studies suggesting that comfortable footwear may serve as a clinical intervention tool rather than merely a means to protect the foot and prevent foot-related diseases (1). In sports medicine, researchers used polarography to measure biomechanical parameters such as peak plantar pressure (PPP) and contact area, analyzing the impact of different types of footwear on foot (2). Measuring plantar pressure is crucial for assessing foot load and predicting the onset and progression of diseases. Pedobarography is the method that enables measurement of pressure between the foot and the shoe during dynamic loading. Pedobarographic measurement systems offer a better understanding of the impact of adjustments in footwear product design on foot mechanics (3, 4) and perceived comfort (5), aiding designers in optimizing footwear products.

With the application of parametric design and additive manufacturing in shoe production, the creation of footwear involves the design of form, lattice structures, and material textures. 3D-printed footwear can reduce material waste, enhance structural stability, and improve wearing comfort (6), catering to consumers' personalized needs (7). Various materials suitable for this manufacturing technology are increasingly used, including thermoplastic polyurethane (TPU), widely applied in the footwear industry. 3D-printed TPU soles are known for their resistance to wear and abrasion and deformation stability. According to the DIN EN ISO 10993-5 and 10993-10 standards, TPU also meets the requirements for cytotoxicity and skin sensitization for medical devices. The primary objective of employing 3D printing technology in shoe soles is to mitigate foot injuries caused by the uneven distribution of plantar pressure and excessive regional pressures and to enhance the comfort of the footwear. However, research on the performance of specific lattice structures, such as auxetic structures in shoe soles, still needs to be completed. Numerous studies have demonstrated the exceptional potential of auxetic materials in sports protection (8, 9). Auxetic structures possess high energy absorption capabilities and superior lightweight characteristics (10). Given the energy absorption and dissipation, compressive and decompressive properties, fatigue toughness, and fracture resistance of auxetic materials (11–13), they present significant potential application value in footwear product design.

**Figure 1:**
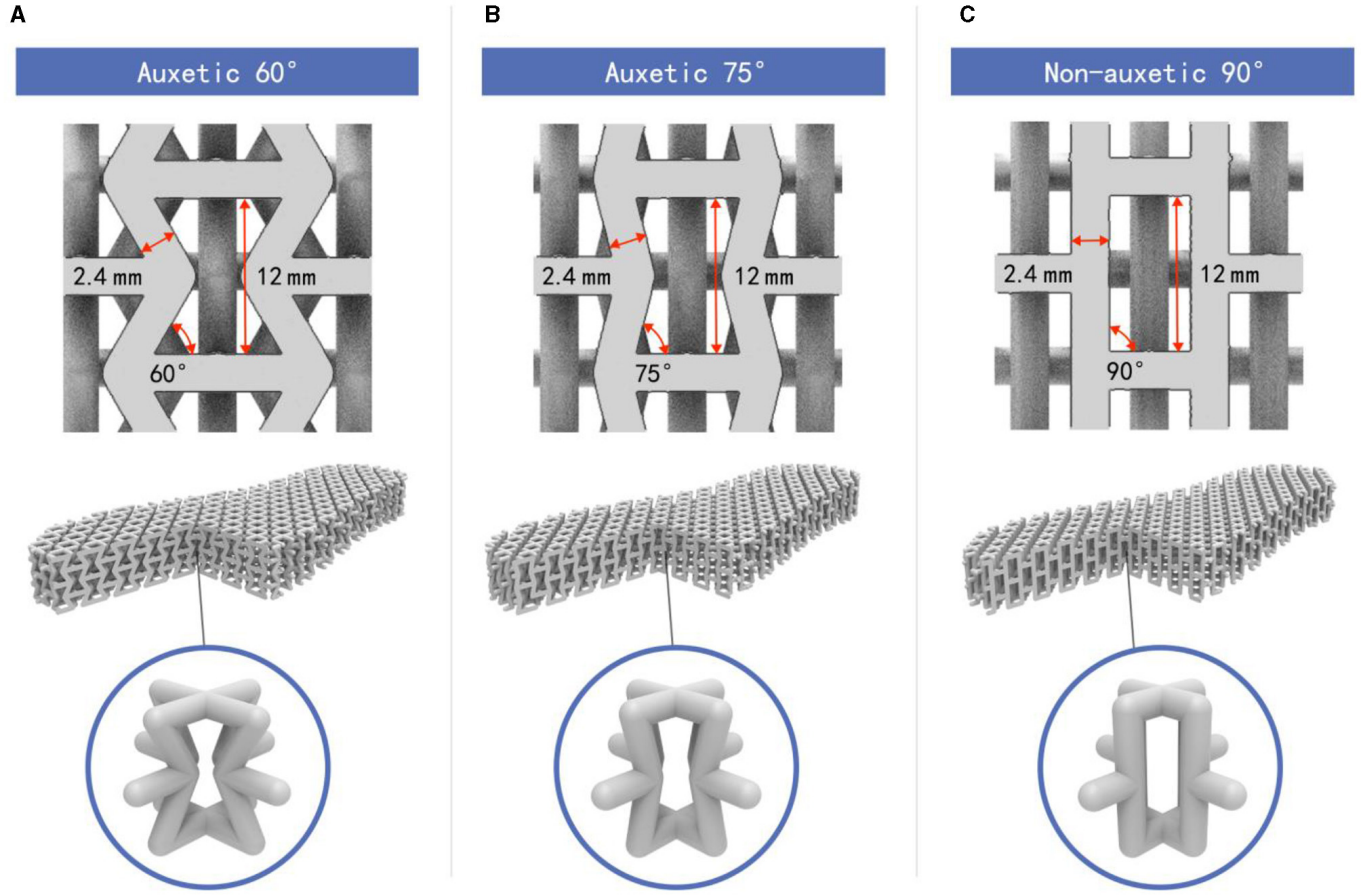
Design of three-dimensional structure and geometric characteristics of each unit cell: **(A)** auxetic 60°, **(B)** auxetic 75°, and **(C)** non-auxetic 90°.

This study explores whether auxetic lattice-structured shoe soles can optimize foot pressure distribution and enhance footwear comfort. The designs of the proposed shoe soles are based on the three-dimensional re-entrant auxetic lattice structure as Figures 1A, B, orthogonally constructed from two-dimensional re-entrant hexagonal honeycombs with internal angles <90 degrees. The material fabricated using this lattice structure exhibits the characteristic of a negative Poisson's ratio, showing expansion laterally under tension and densification under compression. Therefore, such materials are also considered auxetic materials. The research design involves experimental testing of three different lattice-structured shoe soles produced through 3D printing (two auxetic lattice structures with varying internal angles and one non-auxetic lattice structure), with conventional polyurethane (PU) soles as controls. Given the potential differences in the load-bearing functions of the foot during walking and running (14), this study compares the differences in biomechanical parameters and comfort ratings when wearing different soles under both conditions. By exploring the correlation between biomechanical parameters and comfort ratings and identifying physical measurements representing changes in perceived comfort between soles, the study suggests footwear design based on physical causality rather than solely subjective evaluations.

## 2 Footwear comfort, plantar pressure, and contact area

Comfort is among the most crucial evaluation factors for footwear products, primarily measured through subjective assessment methods (15–17). Subjective comfort assessments can be based on a sense of comfort or focus on specific localized areas. Thanks to the development and application of biomechanical sensing devices, researchers have observed parameters such as peak pressures and contact areas at the foot-shoe interface, identifying causes of discomfort when wearing shoes. Consequently, various scientific fields, including sports science, have attempted to explore the relationship between footwear comfort and factors such as plantar surface pressure and foot-shoe contact area, providing a basis and methods for optimizing shoe design.

Some studies have shown a correlation between PPP and footwear comfort. Research by Yung-Hui and Wei-Hsien (18) explored the effects of shoe inserts and heel height on the plantar pressure during women's walking and found significant correlations between peak pressures in the medial forefoot and midfoot regions and evaluations of plantar comfort. Mei et al. revealed that the measurements of peak plantar pressures (PPPs), in the first metatarsal head, medial midfoot, and hindfoot regions, are closely related to the comfort of the footwear in long-distance running tests (19). A study on the role of insoles with various wedges during marching revealed a negative correlation between PPP, in second and third metatarsal head, and the midfoot regions, and perceived plantar comfort (20). However, some studies have shown that the impact of PPPs on the assessment of footwear comfort is minimal (21, 22) or even nonexistent (23, 24). Numerous studies have sought to identify the relationship between overall or localized PPP measurements and subjective self-reported comfort ratings, yet results remain primarily inconsistent. However, factors such as age, gender, and BMI that cause differences in bodily sensitivity may influence subjective comfort ratings (25), such as a broad age range of participants or mixed test result data from male and female participants, leading to varied analysis outcomes.

In ergonomics-related research, the relationship between the contact area of body-touching parts and subjective comfort ratings is frequently discussed, particularly in the context of products such as seat cushions (26, 27), exoskeletons (28), and pillows (29). Goonetilleke (30) posited that within the maximum pressure tolerance range, the contact area of a region correlates positively with perceived comfort. Research on footwear products has also consistently discussed the role of plantar contact areas in the comfort of shoes. For instance, the comfort of casual shoes may be related to the contact area in the midfoot region (31). Similarly, studies on high-heel shoe sole morphology have indicated a close relationship between contact area and perceived comfort (32), suggesting that increasing the contact area between the insole and the arch through extra mid-foot support can enhance footwear comfort (33). Focusing on the foot structure changes in early-old adults, Puszczalowska-Lizis, Koziol (34) noted that designing shoes with an appropriate profile and construction can increase the contact area between the foot and the shoe, effectively improving comfort perception for individuals with specific foot shapes. Increasing the contact area between the plantar surface and the insole may enhance the perception of plantar comfort.

Therefore, the study compares the results of four types of shoe soles to understand the impact of peak pressure and contact area on different plantar regions on the perception of comfort, providing a reference for the comfort design of shoe soles with auxetic structure.

## 3 Methods

### 3.1 Participants

This study recruited 20 healthy male volunteers, all customarily wearing EU-size 42 shoes. The criteria for participant inclusion were as follows: (1) no history of lower limb fractures or surgeries; (2) absence of pain or discomfort while walking; (3) no deformities such as hallux valgus, flat feet, or bow legs in the lower limbs; and (4) normal walking posture. Based on these criteria, participants aged between 19 and 23 years old, with an average height of (171 ± 1.9) cm, average mass of (69 ± 4.6) kg, and foot length of (253 ± 1.0) mm.

### 3.2 Design and fabrication of the shoe soles

The study compared four shoe soles comprising two material types: shoe soles with a three-dimensional lattice structure made of TPU and commonly used Polyurethane (PU) shoe soles as controls. The TPU shoe soles featured three different lattice structures: auxetic lattice structures with internal angles of 60° and 75° and a non-auxetic structure with an internal angle of 90° (Figure 1). This lattice structure adopts the typical auxetic lattice structure proposed by Evans, et al.—the re-entrant hexagonal honeycomb structure (35), the characteristics of which are still being explored in various fields (36). According to the analytical model described in the study by Evans et al. the Poisson's ratio is negative when the internal angle of the hexagon is <90°, which ensures that the samples with internal angles of 60° and 75° in this study can exhibit auxetic behavior. Due to the softness of the TPU material and the limitations of FDM 3D printing with TPU material, collapse often occurs when the angle between the ribs on both sides of the lattice unit and the horizontal plane is <60° (i.e., internal angle <60°), making sample fabrication impossible. Therefore, the minimum internal angle of the lattice structure was set to 60° in the experiments. The three-dimensional structure and geometric characteristics of each unit cell are illustrated in Figure 1. Each lattice unit featured ribs with a diameter of 2.4 mm and a height of 12 mm. Based on multiple attempts at sample fabrication, it is determined that setting the rib height of the lattice unit to 12 mm provides sufficient space for lattice deformation when the shoe sole is under compression. The lattices within the shoe soles were uniformly arranged, with each sole comprising two units of lattices arranged longitudinally. The study aimed to control the volume of the lattice structure in the three 3D printing shoe soles as consistently as possible (material volume of 124.84 ± 0.88 cm ∧ 3), maintaining a similar volume-to-total space ratio (approximately 22.92%) to minimize the impact of relative density on the mechanical properties of the shoe soles. The 3D models of the lattice-structured elastic shoe soles were constructed using Rhino 7^®^ and Grasshopper^®^ 3.5 software. Subsequently, the A60, A75, and N90 shoe soles were fabricated using Fused Deposition Modeling (FDM) 3D printing technology.

**Figure 2:**
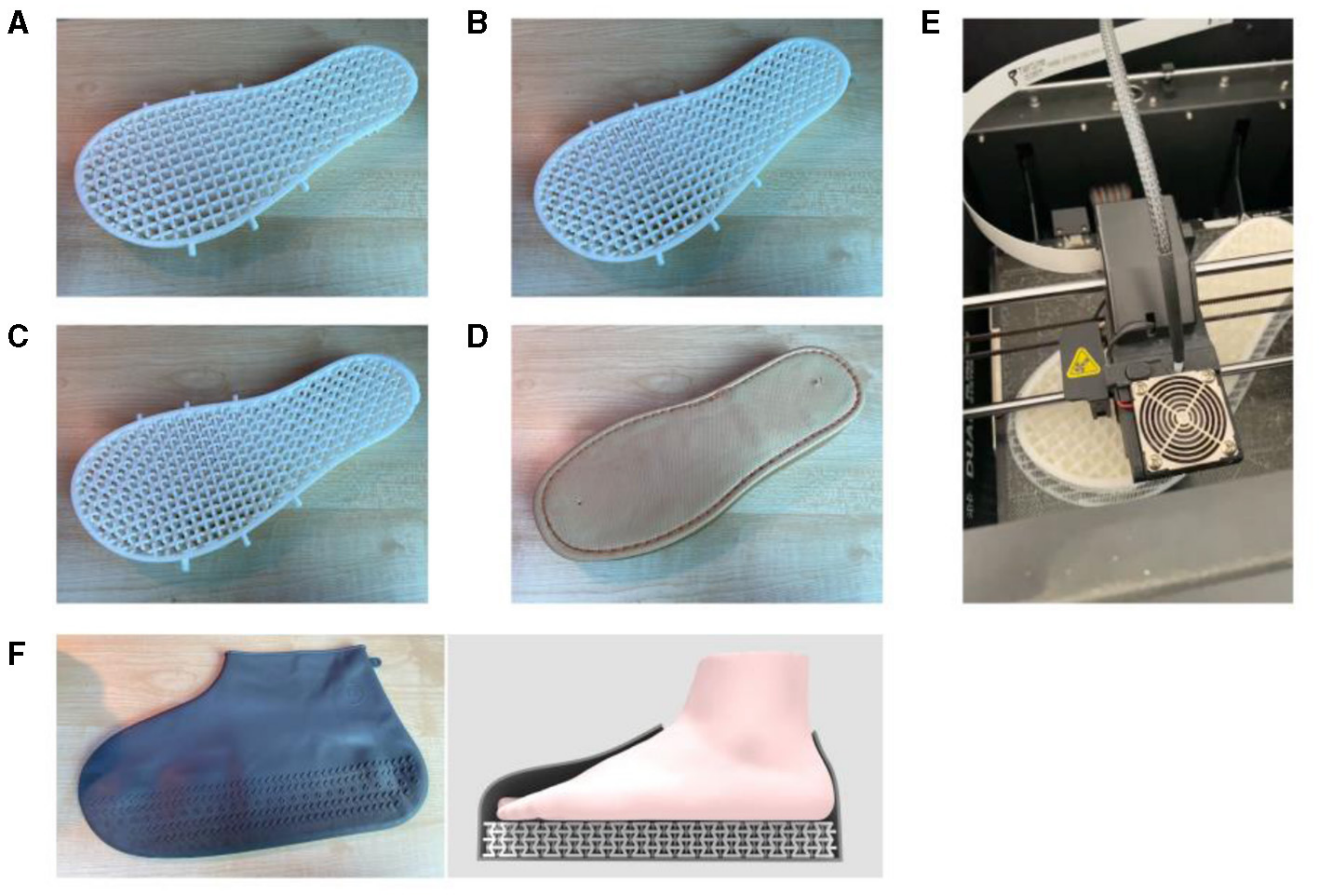
The four different types of shoe soles used in this study: **(A)** A60, **(B)** A75, **(C)** N90, **(D)** PU, **(E)** 3D printing process and **(F)** the rubber overshoe used to secure the soles.

This study utilized the UP300 model 3D printer from Tiertime (China, Beijing) for sample fabrication, with the shoe sole models designed and imported into the printer using the configured UP Studio software. The nozzle displacement precision of the device on the x, y, and z axes is 2, 2, and 0.5 micrometers, respectively. The printing accuracy is 0.1 MM. The printing process involves setting the layer thickness to 0.2 mm and the print quality to the highest setting. As shown in Figures 2A–C, each sample to compared with PU shoe sole as Figure 2D, was printed using durable and elastic 95A TPU material. The FDM 3D printing process is shown as Figure 2E.

### 3.3 Procedures

Participants were required to walk and run 20 meters wearing each of the four types of shoe soles. During the experiment, each shoe sole was secured to the foot by a rubber shoe cover, as shown in Figure 2F. The experiments were conducted on a straight and flat track to effectively collect biomechanical data from various regions of the plantar foot. However, through multiple experimental attempts, it was found that beyond a distance of 10 meters between the data collector and the signal receiver, the wireless signal becomes unstable, leading to potential data loss. Therefore, the signal receiver was placed in the middle of the track, and the track distance was set to 20 meters, exceeding the 10-meter walkway tested in a study by Chatrenet et al. (37) and the 16-meter runway tested in a study by Hamzavi and Esmaeili (38). Therefore, the study considers a distance of 20 meters sufficient to measure plantar pressure distribution during walking and running effectively. Since walking and running speeds can influence plantar pressure and ground reaction forces (39–41), the experiment required the participants to warm up on a treadmill for 3 min prior to walking and running tests, with walking speeds set at 110 cm/s and running speeds at 280 cm/s. After becoming accustomed to the walking and running speeds, participants conducted the plantar pressure system experiment and subjective plantar comfort ratings. A 3-min rest was allotted between each test to prevent fatigue. Finally, the study obtained 160 valid experimental results (20 subjects, 4 types of soles, and 2 modes of movement), each including plantar pressure data, foot-shoe contact area, and plantar comfort ratings for subsequent data analysis.

### 3.4 Pedobarographic measurements

**Figure 3:**
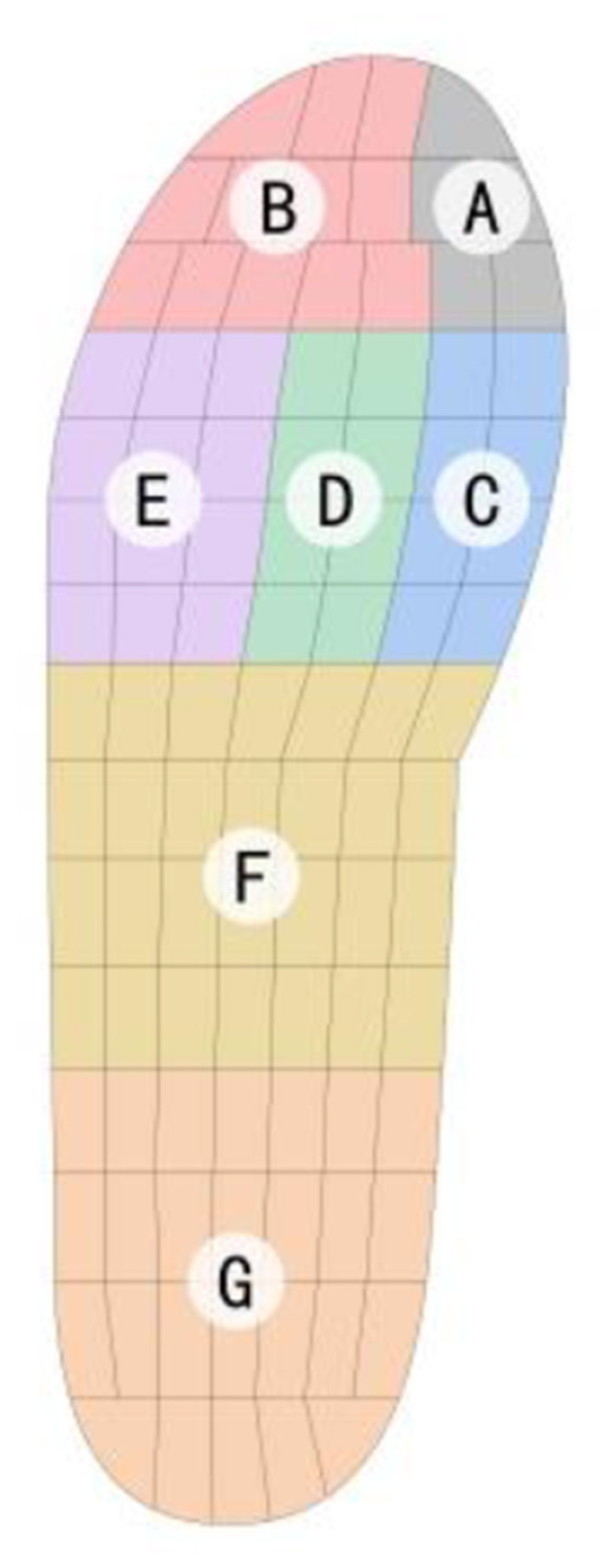
Plantar regions selected for measuring peak pressure and contact area: **(A)** Hallux. **(B)** Second–fifth toe (Toes 2–5). **(C)** First metatarsal head (MTH 1). **(D)** Second metatarsal head (MTH 2). **(E)** Third–fifth metatarsal head (MTH 3–5). **(F)** Midfoot. **(G)** Hindfoot.

The Pedar-X in-shoe pressure measurement system (Novel Co., Munich, Germany) was utilized to collect plantar pressure data. The Pedar insole, insole-shape data collector of the system, contains 99 pressure sensors with a pressure range of 15–1,200 kPa placed below the foot. One side of the insole is connected to the A/D conversion electronics worn around the waist by a data cable. Plantar pressure data were wirelessly transmitted to a connected computer through the A/D conversion electronics, ensuring the measurement process did not interfere with gait characteristics. The reliability and effectiveness of the Pedar-X system have been verified in multiple studies (18). The experiment used the Pedar insole sized 42/43 EU with a thickness of 1.9 mm and recorded foot pressures at a frequency of 50 Hz. Before testing, the experiment divided the plantar foot into seven regions in the pressure measurement system: the hallux, toes 2-5, metatarsal heads (MTH) 1, MTH 2, MTH 3-5, midfoot, and hindfoot (Figure 3). After testing, the Pedar-X system software (Pedar online) calculated the PPP and contact area across plantar regions during the gait cycle.

### 3.5 Subjective comfort measurements

Participants rated the comfort of their feet on a 10 cm visual analog scale (VAS) after each experimental test. Participants evaluated not only the overall comfort of the foot but also the comfort of specific areas (toe area, forefoot, and heel). This study's VAS, adapted from Bousie, Blanch (15), was anchored with “not comfortable at all” (0) and “most comfortable imaginable” (10). The experiment required participants to evaluate only the comfort of the plantar surface, excluding characteristics such as the appearance and breathability of the overshoe from the assessment of plantar comfort.

### 3.6 Data analysis

The test result data were first subjected to Shapiro-Wilk and Kolmogorov-Smirnov tests to confirm normality analysis. Subsequently, one-way ANOVA was used to study the effects of different types of shoe soles on plantar pressure. Tukey's HSD test was employed for *post hoc* comparison to evaluate pairwise differences among the four tested shoe soles. A *p* < 0.05 was set for statistical significance. Pearson correlation coefficients were calculated to evaluate the relationship between comfort ratings and variables of PPP and contact area. All statistical analyses were conducted using IBM SPSS ver. 23.0 (IBM Corp., Armonk, NY, USA).

## 4 Results

### 4.1 Peak plantar pressures

**Figure 4:**
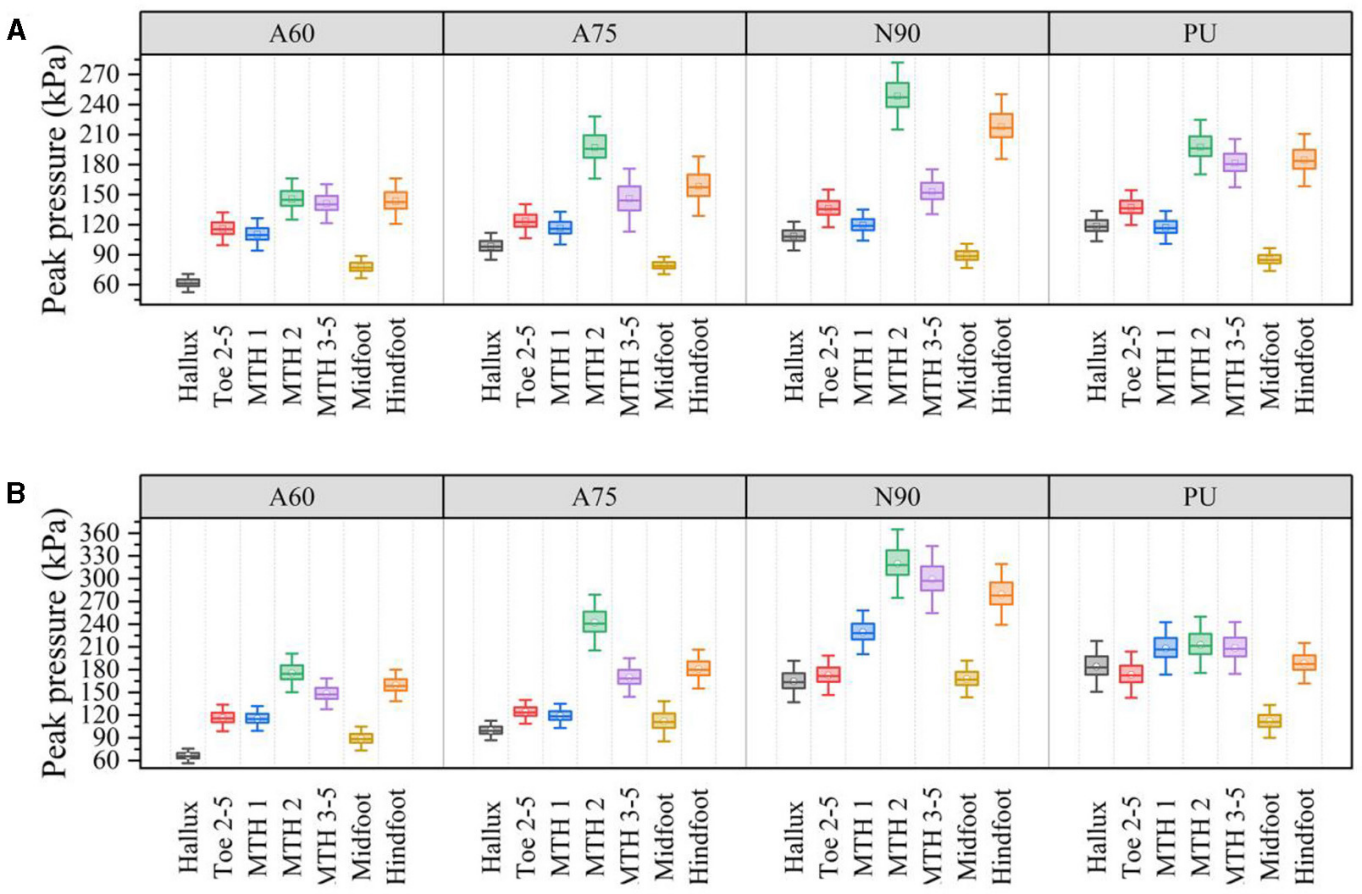
PPPs in different plantar regions with four different types of shoe soles during walking and running. **(A)** Walking. **(B)** Running.

Figure 4 compares PPPs in different plantar regions of the foot while wearing various shoe soles in walking and running states.

The F-value is one of the most critical statistics in ANOVA, representing the ratio of between-group differences to within-group differences. If the F-value exceeds the critical F-value (2.70), it indicates differences between the groups. A higher F-value suggests more significant differences between groups. The study validated differences in intergroup (A60\A75\N90\PU) data, demonstrating the influence of shoe sole lattice structures on pressure in various plantar regions. Based on the ANOVA results, the type of shoe soles had a significant effect on the peak pressures in the hallux [F_(3, 76)_ = 234.5, *p* < 0.001], Toe 2-5 [F_(3, 76)_ = 23.0, *p* < 0.001], MTH 2 [F_(3, 76)_ = 143.7, *p* < 0.001], MTH 3-5 [F_(3, 76)_ = 33.2, *p* < 0.001], midfoot [F_(3, 76)_ = 15.4, *p* < 0.001], and hindfoot [F_(3, 76)_ = 90.2, *p* < 0.001] regions of the foot during walking. *Post hoc* comparisons using Tukey's HSD test revealed that auxetic structured shoe soles (A60 and A75) significantly reduced peak pressures in most plantar regions (including hallux, Toe 2-5, MTH 2, midfoot, and hindfoot) during walking compared to the non-auxetic structured shoe sole N90. Additionally, for running, shoe soles' type significantly affected the peak pressures in the hallux [F_(3, 76)_ = 378.0, *p* < 0.001], Toe 2-5 [F_(3, 76)_ = 114.8, *p* < 0.001], MTH 1 [F_(3, 76)_ = 363.9, *p* < 0.001], MTH 2 [F_(3, 76)_ = 182.7, *p* < 0.001], MTH 3-5 [F_(3, 76)_ = 280.4, *p* < 0.001], midfoot [F_(3, 76)_ = 147.7, *p* < 0.001], and hindfoot [F_(3, 76)_ = 218.0, *p* < 0.001] regions. *Post hoc* comparisons using Tukey's HSD test also indicated that auxetic structured shoe soles (A60 and A75) significantly lowered peak pressures in all plantar regions during running compared to the non-auxetic structured shoe sole N90. Table 1 compares the mean values of peak pressures in various foot regions for different shoe soles and calculates the differences in mean comparisons, with *p*-values indicating the significance of pairwise comparison results of sample means. The percentages in brackets in Table 1 represent the extent to which the pressure in the first sample decreases compared to the second sample in paired comparisons. According to Table 1, for both walking and running conditions, 3D-printed lattice shoe soles with auxetic structures significantly (or non-significantly) reduced peak pressures in various plantar regions compared to non-auxetic shoe soles, optimizing foot pressure distribution. Among them, A60 significantly reduced peak pressures in the hallux, MTH 2, and hindfoot regions during walking and significantly reduced peak pressures in the hallux, MTH 2, MTH 3-5, midfoot, and hindfoot regions during running. Therefore, it can be inferred that the auxetic A60 sole exhibits superior pressure reduction capabilities.

**Table 1:** The mean differences in PPPs between four different types of shoe soles.

**Variable**	**A60-A75**	**A60–N90**	**A60–PU**	**A75–N90**	**A75–PU**	**N90–PU**
**Walking**						
Hallux	−36.718^***^	−46.977^***^	−56.910^***^	−10.259^***^	−20.192^***^	−9.934^***^
	(37.34%)	(43.26%)	(48.02%)	(9.45%)	(17.04%)	(8.38%)
Toes 2-5	−7.441	−20.268^***^	−21.151^***^	−12.827^***^	−13.710^***^	−0.883
	(6.03%)	(14.89%)	(15.43%)	(9.42%)	(10.00%)	(0.64%)
MTH 1	−6.208	−9.291^**^	−6.947	−3.084	−0.0740	2.344
	(5.33%)	(7.78%)	(5.93%)	(2.58%)	(0.63%)	(2.00%)
MTH 2	−51.422^***^	−102.862^***^	−51.799^***^	−51.400^***^	−0.377	51.064^***^
	(26.11%)	(41.41%)	(26.25%)	(20.71%)	(0.19%)	(25.88%)
MTH 3-5	−5.051	−11.484^*^	−40.441	−6.798	−35.390^***^	−28.593^***^
	(3.46%)	(7.75%)	(22.29%)	(4.45%)	(19.51%)	(15.76%)
Midfoot	−1.665	−11.334^***^	−7.648^**^	−9.669^***^	−5.984^*^	3.686
	(2.10%)	(12.76%)	(8.98%)	(10.89%)	(7.03%)	(4.33%)
Hindfoot	−14.942^*^	−74.429^***^	−41.043^***^	−59.488^***^	−26.102^***^	33.386^***^
	(9.44%)	(34.17%)	(22.25%)	(27.31%)	(14.15%)	(18.10%)
**Running**						
Hallux	−33.678^***^	−98.257^***^	−118.158^***^	−64.579^***^	−84.480^***^	−19.901^***^
	(33.77%)	(59.80%)	(64.14%)	(39.30%)	(45.86%)	(10.80%)
Toes 2-5	−7.845	−56.179^***^	−56.974^***^	−48.334^***^	−49.129^***^	−0.796
	(6.32%)	(32.57%)	(32.88%)	(28.03%)	(28.36%)	(0.46%)
MTH 1	−3.519	−113.773^***^	−92.456^***^	−110.254^***^	−88.937^***^	−21.317^***^
	(2.96%)	(49.64%)	(44.48%)	(48.11%)	(42.79%)	(10.26%)
MTH 2	−66.520^***^	−144.069^***^	−37.112^***^	−77.549^***^	29.408^***^	106.957^***^
	(27.49%)	(45.08%)	(17.46%)	(24.27%)	(13.83%)	(50.31%)
MTH 3-5	−21.489^**^	−150.659^***^	−60.521^***^	−129.170^***^	−39.032^***^	90.138^***^
	(12.68%)	(50.44%)	(29.02%)	(43.25%)	(18.72%)	(43.23%)
Midfoot	−22.814^***^	−78.660^***^	−22.661^***^	−55.847^***^	0.153^***^	55.999^***^
	(20.41%)	(46.93%)	(20.30%)	(33.32%)	(0.14%)	(50.17%)
Hindfoot	−21.496^***^	−119.867^***^	−29.177^***^	−98.371^***^	−7.681	90.691^***^
	(11.90%)	(42.95%)	(15.49%)	(35.25%)	(4.08%)	(48.14%)

^*^the mean difference is significant at the 0.05 level.

^**^the mean difference is significant at the 0.01 level.

^***^the mean difference is significant at the 0.001 level.

### 4.2 Contact area

Furthermore, ANOVA analysis revealed that the sole type significantly affected the contact area in various plantar regions during walking and running (*p* < 0.01). Table 2 compares the mean values of contact areas in various foot regions for different shoe soles and calculates the differences in mean comparisons, with *p*-values indicating the significance of pairwise comparison results of sample means. *Post hoc* comparisons presented in Table 2 demonstrated that auxetic structured shoe soles (A60 and A75) significantly increased the contact area in the MTH 1, midfoot and hindfoot regions during walking compared to the non-auxetic A90 shoe sole and significantly increased the contact area in the hallux, MTH 1, MTH 2, MTH 3-5, and midfoot regions during running. Among the auxetic structures, A60 showed a significantly larger contact area in the Toe 2-5, MTH 1, and MTH 3-5 regions during walking and all metatarsal heads of the forefoot regions during running, compared to A75. Thus, it can be inferred that the internal angles within the auxetic concave structures influence the deformation of lattice units and the contact area across different regions of the plantar surface. Compared to the PU shoe sole, the A60 shoe sole significantly increased the foot-shoe contact area in all plantar regions while walking and running.

**Table 2:** Mean differences in contact areas (cm^2^) between four different types of shoe soles.

**Variable**	**A60-A75**	**A60-N90**	**A60-PU**	**A75-N90**	**A75-PU**	**N90-PU**
**Walking**						
Hallux	0.001	0.206	1.096^***^	0.205	1.095^***^	0.891^***^
Toes 2-5	2.160^***^	3.370^***^	4.353^***^	1.210^**^	2.194^***^	0.984^*^
MTH 1	1.721^***^	3.452^***^	3.762^***^	1.731^***^	2.041^***^	0.310
MTH 2	0.503	1.022^***^	1.042^***^	0.519	0.539	0.020
MTH 3-5	2.030^***^	2.490^***^	3.023^***^	0.460	0.993^**^	0.533
Midfoot	0.806	5.989^***^	9.093^***^	5.183^***^	8.287^***^	3.104^***^
Hindfoot	1.597	6.913^***^	6.694^***^	5.317^***^	5.098^***^	−0.219
Running						
Hallux	0.099	1.795^***^	2.092^***^	1.696^***^	1.993^***^	0.298
Toes 2-5	0.058	0.465	1.102^***^	0.408	1.045^***^	0.637
MTH 1	0.773^**^	1.805^***^	2.189^***^	1.032^***^	1.416^***^	0.384
MTH 2	0.740^**^	1.510^***^	1.903^***^	0.770^**^	1.163^***^	0.393
MTH 3-5	1.727^***^	2.509^***^	3.103^***^	0.782^*^	1.376^***^	0.594
Midfoot	1.685	5.341^***^	6.384^***^	3.656^***^	4.699^***^	1.043
Hindfoot	0.676	2.797	3.737^**^	2.121	3.061^*^	0.940

^*^the mean difference is significant at the 0.05 level.

^**^the mean difference is significant at the 0.01 level.

^***^the mean difference is significant at the 0.001 level.

### 4.3 Plantar comfort ratings

**Figure 5:**
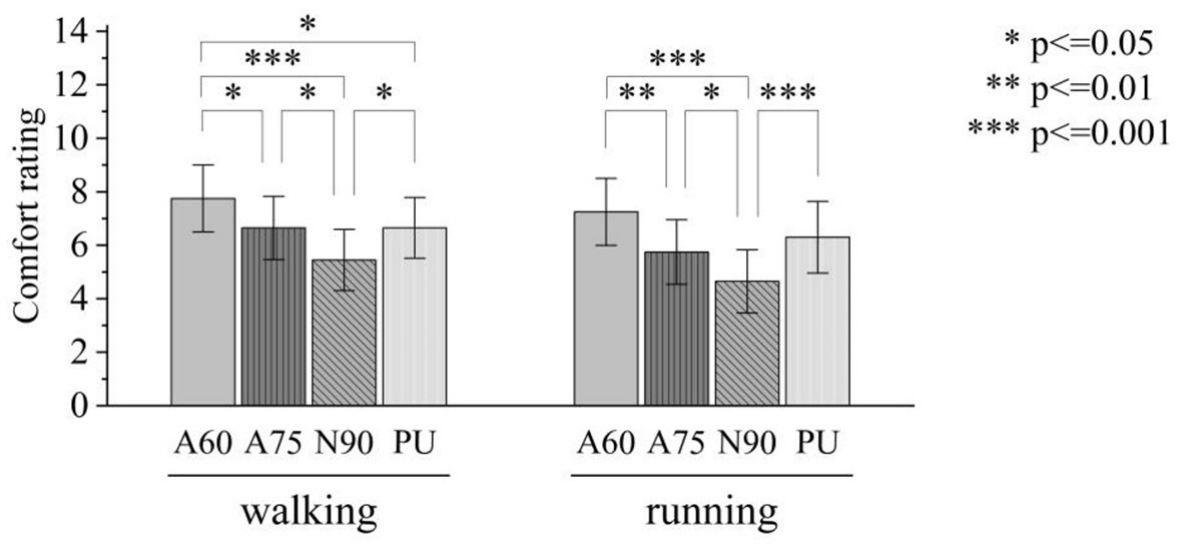
Comparisons of comfort ratings for each test condition.

Figure 5 illustrates the comparisons of comfort ratings for each test condition. ANOVA results indicated that the type of shoe sole had a significant effect on comfort ratings not only during walking [F_(3, 76)_ = 12.7, *p* < 0.01] but also during running [F_(3, 76)_ = 15.2, *p* < 0.01]. Post hoc comparisons revealed that, in both walking and running scenarios, the comfort ratings of auxetic shoe soles (A60 and A75) were significantly higher than those of the non-auxetic N90 shoe soles within the 3D printed shoe soles. Furthermore, the comfort of the A60 shoe sole was significantly higher than that of the A75 shoe sole. In walking scenarios, the comfort of the A60 shoe sole was also significantly higher than that of the PU shoe sole.

Table 3 lists the coefficients of correlation between measures of biomechanical variables and comfort ratings. Under the condition of significant correlation (*p* < 0.05), higher absolute values of the correlation coefficients in Table 3 indicate a stronger relationship between comfort ratings and peak pressure or contact area in specific foot regions. The positive or negative values indicate the positive or negative impact of peak pressure or contact area on comfort ratings. From Table 3, peak pressure in various foot regions negatively impacts the comfort ratings, while contact area positively impacts the comfort ratings. In walking state, comfort rating showed a significant negative correlation with peak pressures in the Hallux (−0.327), MTH 2 (−0.647), and Hindfoot (−0.359) regions. In running state, comfort rating was also significantly negatively correlated with peak pressures in the MTH 2 (−0.337), MTH 3-5 (−0.322), Midfoot (−0.285), and Hindfoot (−0.316) regions. However, comfort ratings were positively correlated with the contact area in various plantar regions at a significance level of 0.01, regardless of whether walking or running.

**Table 3:** Coefficients of correlation between measures of biomechanical variables and comfort ratings.

**Variables**	**Correlation coefficient**		**Variables**	**Correlation coefficient**	
	**Walking**	**Running**		**Walking**	**Running**
**Peak pressure by regions**			**Contact area by regions**		
Hallux	−0.327^**^	−0.183	Hallux	0.312^**^	0.381^**^
Toes 2-5	−0.155	−0.048	Toes 2-5	0.586^**^	0.321^**^
MTH 1	−0.004	−0.215	MTH 1	0.590^**^	0.475^**^
MTH 2	−0.647^**^	−0.337^**^	MTH 2	0.637^**^	0.477^**^
MTH 3-5	0.053	−0.322^**^	MTH 3-5	0.619^**^	0.502^**^
Midfoot	−0.156	−0.285^*^	Midfoot	0.479^**^	0.466^**^
Hindfoot	−0.359^**^	−0.316^**^	Hindfoot	0.636^**^	0.395^**^

^**^correlation is significant at the 0.01 level. ^*^correlation is significant at the 0.05 level.

## 5 Discussion

The results of this experiment support our hypothesis that auxetic structured shoe soles (A60 and A75) reduce PPPs more effectively than non-auxetic soles (N90). In specific movement states, effectively reducing the peak pressures across different areas of the plantar surface indicates a better distribution of foot pressure (42). According to Table 1, the *post hoc* comparisons showed that the peak pressure mean differences between different 3D-printed shoe soles were significant across various plantar regions. Under walking conditions, A75 reduced the pressure by 9.42% to 27.31% compared to N90, and A60 reduced it by 7.75% to 43.26% compared to N90. Under running conditions, A75 reduced the pressure by 24.27% to 48.11% compared to N90, and A60 reduced it by 32.57% to 59.80% compared to N90. These findings align with existing research on the energy absorption capabilities (8–10) and auxetic materials' pressure reduction characteristics (43). However, due to the inherent rigidity of the TPU material, only the auxetic A60 sole demonstrated better pressure reduction across all regions of the plantar surface compared to the existing PU soles in this study. Referring to the mean differences and *post hoc* comparisons of peak pressure between the A60 and PU across various plantar regions, the A60 shoe sole effectively reduced pressures by 8.98% to 48.02% under walking conditions and by 15.49% to 64.14% under running conditions.

Furthermore, results from Table 2 indicate that the plantar contact area for auxetic soles (A60 and A75) is generally more significant than that for non-auxetic soles (N90 and PU). Notably, the A60 shoe sole showed a significant increase in the contact area across all regions of the plantar surface compared to the PU shoe sole. A higher contact area confirms that soles with an auxetic lattice structure conform better to the plantar shape (44). In particular, shoe soles with an internal angle of 60° auxetic lattice structure exhibit higher conformability and adaptability to the plantar surface (45). Consistent with current viewpoints on the application of auxetic structures, auxetic materials can conform to curved surfaces through the formation of synclastic curvature, such as the human body surface (46). Existing research has demonstrated that personal protective equipment with auxetic elements embedded in sports apparel offers better fit and comfort (44) and more effectively prevents bodily injuries (8) than non-auxetic alternatives. By increasing contact area, the form-fitting characteristics of auxetic lattice structures can effectively uniformize pressure distribution and reduce peak pressures. Based on this principle, robotic grippers employing auxetic structures have seen enhanced stability in grasping objects (47). Similarly, shoe soles utilizing auxetic lattice structures are better able to conform to the contours of the plantar surface, increasing the force distribution area on the foot, thereby improving plantar pressure distribution and reducing peak pressures.

Evidence suggests that shoe sole designs that reduce pressure and enhance comfort are paramount in maintaining foot health (7, 48, 49), revealing several potential applications of auxetic lattice structures in various types of shoe soles in the future. Table 3 shows a correlation between peak pressures in certain plantar regions and the perception of comfort. Identifying correlations between comfort ratings and the measurements of peak pressures in different plantar regions can provide references for optimizing sole design. Based on the results of correlation analysis (Table 3), the A60 auxetic structured shoe sole proposed in this study aids in improving footwear comfort and ensuring foot health, especially when applied to the sole areas of the hallux, MTH 2, and hindfoot in casual everyday footwear, and the MTH 2, MTH 3-5, midfoot, and hindfoot areas in running shoes. For instance, previous studies have shown that females tend to exert more pressure on the front and medial side of the foot during walking (50), causing higher pressure in the Hallux region and increasing the likelihood of developing Hallux Valgus (HV) (51).

Consequently, the study posits that footwear applying auxetic 60° lattice structures in the shoe sole be a potential method to prevent HV in females, as Martínez-Nova, Sánchez-Rodríguez (52) suggested, by relieving plantar pressure in the forefoot and Hallux region. Metatarsalgia is often caused by pressures exceeding the pressure tolerance of the tissue beneath the metatarsal heads due to factors such as tight ankle plantar flexors, wearing high-heeled shoes, claw- or hammertoes, and dysfunction of the first metatarsal phalangeal joint (53). Existing solutions primarily include custom insole shapes or placing metatarsal pads on the sole (54), or employing the Hunt Metatarsal External Shoe Cut-out (HMESC) method (54), which are challenging to implement in mass production. Therefore, Utilizing the A60 shoe sole may represent a potential means to alleviate metatarsalgia while also being amenable to mass production. Moreover, given the outstanding pressure-relieving performance of the A60 shoe sole in the heel area, it could also apply to diabetic foot treatment shoes, footwear for old age, or orthotic shoes with hindfoot relief functions (4). The efficiency of shock absorption in the heel tissues of diabetic patients and the suppression of gait impact forces are diminished, making the protection of heel tissues in diabetic patients and the prevention of diabetes-related foot ulceration (DFU) a long-standing significant topic in footwear product optimization research (55, 56). Similarly, many older adult individuals experience hyperkeratosis and region-specific foot pain due to the loss of elastin and collagen fibers in the plantar surface (57). To avoid overloading damage to bones and soft tissues or to cause adverse sequelae (e.g., ulcers, gait abnormalities) in pre-existing tissue disabilities, shoe soles must also reduce pressure and protect the heels of old age. The A60 lattice structure could meet the shock-absorbing design requirements of running shoes by attenuating peak pressures in the heel and forefoot regions, increasing the contact area in the midfoot, and thereby reducing the likelihood of calf injuries during running (58).

## 6 Conclusion

This study introduces the concept of incorporating auxetic lattice structures into shoe sole design. Experimental findings confirm that such integration effectively enhances pressure-reducing performance and conformity of the sole to the foot, consequently improving plantar comfort. The study evaluated plantar biomechanical parameters (peak pressures and contact areas across various regions of the foot) and comfort by experimentally comparing auxetic lattice soles (A60 and A75), non-auxetic lattice soles (N90), and conventional PU soles in walking and running states. As result, auxetic lattice structured shoe soles (A60 and A75) are more effective in reducing plantar pressure compared to non-auxetic N90. Additionally, the plantar contact area is generally more substantial for auxetic soles (A60 and A75) than for non-auxetic soles (N90 and PU). Based on experimental data, the study analyzed the correlation between plantar biomechanical parameters and comfort ratings. In both walking and running states, comfort ratings exhibit significant negative correlations with peak pressures in specific foot regions, including the Hallux, MTH 2, MTH 3-5, Midfoot, and Hindfoot, while demonstrating consistent positive correlations with contact area across various plantar regions. The correlation analysis between plantar parameters and comfort ratings provides significant references for optimizing sole structures. Furthermore, leveraging the exceptional characteristics of auxetic structured shoe soles, the study discusses the potential applications of auxetic design structured shoe soles (A60) in preventing foot diseases such as hallux valgus, metatarsalgia, and diabetic foot.

Although the study has gained valuable insights through rigorous experimental research, some limitations remain. The study exclusively recruited male participants for experiments and only conducted short-term mechanical performance tests of the soles. To further refine the design of auxetic structured shoe soles, it is imperative to incorporate gender factors into the experiments and conduct long-term experiments on the specific footwear design with auxetic lattice structure in future research.

## Data availability statement

The raw data supporting the conclusions of this article will be made available by the authors, without undue reservation.

## Ethics statement

The studies involving humans were approved by Research Management Department at Guangdong University of Technology. The studies were conducted in accordance with the local legislation and institutional requirements. The participants provided their written informed consent to participate in this study.

## Author contributions

JZ: Conceptualization, Investigation, Validation, Writing – original draft. SL: Funding acquisition, Project administration, Supervision, Writing – review & editing. YY: Data curation, Formal analysis, Methodology, Writing – review & editing. YL: Formal analysis, Software, Validation, Writing – original draft. YG: Data curation, Formal analysis, Software, Writing – original draft. HW: Investigation, Resources, Visualization, Writing – original draft.

## References

[1] Menz HB Bonanno DR. Footwear comfort: a systematic search and narrative synthesis of the literature. J Foot Ankle Res. (2021) 14:1–11. 10.1186/s13047-021-00500-9 34876192 PMC8650278

[2] Cho YJ Lee D-W Shin HS Hwang YB Lee DO Kim D-Y. Change of in-shoe plantar pressure according to types of shoes (flat shoes, running shoes, and high heels). Clin Orthop Surg. (2022) 14:281. 10.4055/cios20260 35685969 PMC9152888

[3] Caravaggi P Giangrande A Lullini G Padula G Berti L Leardini A. In shoe pressure measurements during different motor tasks while wearing safety shoes: the effect of custom made insoles vs. prefabricated and off-the-shelf. Gait Posture. (2016) 50:232–8. 10.1016/j.gaitpost.2016.09.013 27662483

[4] Mazur F Swoboda B Carl H Lutter C Engelhardt M Hoppe M. Plantar pressure changes in hindfoot relief devices of different designs. J Exp Orthopaed. (2019) 6:1–8. 10.1186/s40634-019-0173-9 30729337 PMC6367492

[5] Zwaferink JB Custers W Paardekooper I Berendsen HA Bus SA. Optimizing footwear for the diabetic foot: data-driven custom-made footwear concepts and their effect on pressure relief to prevent diabetic foot ulceration. PLoS ONE. (2020) 15:e0224010. 10.1371/journal.pone.0224010 32324739 PMC7179916

[6] Cui T. Disruption in Digital Fabrication: Exploring FDM 3D Printing and 3D CAD for a Wearable Apparel Product. Auburn: Auburn University (2019).

[7] Zolfagharian A Lakhi M Ranjbar S Bodaghi M. Custom shoe sole design and modeling toward 3D printing. Int J Bioprint. (2021) 7:396. 10.18063/ijb.v7i4.396 34805590 PMC8600303

[8] Duncan O Shepherd T Moroney C Foster L Venkatraman PD Winwood K. Review of auxetic materials for sports applications: expanding options in comfort and protection. Appl Sci. (2018) 8:941. 10.3390/app8060941

[9] Allen T Shepherd J Hewage T Senior T Foster L Alderson A. Low-kinetic energy impact response of auxetic and conventional open-cell polyurethane foams. Physica Status Solidi. (2015) 252:1631–9. 10.1002/pssb.201451715

[10] Han D Ren X Zhang Y Zhang XY Zhang XG Luo C. Lightweight auxetic metamaterials: Design and characteristic study. Composite Struct. (2022) 293:115706. 10.1016/j.compstruct.2022.115706

[11] Prawoto Y. Seeing auxetic materials from the mechanics point of view: a structural review on the negative Poisson's ratio. Comp Mater Sci. (2012) 58:140–53. 10.1016/j.commatsci.2012.02.012

[12] Zhang Y Sun L Ren X Zhang XY Tao Z Xie YM. Design and analysis of an auxetic metamaterial with tuneable stiffness. Comp Struct. (2022) 281:114997. 10.1016/j.compstruct.2021.114997

[13] Dong Z Li Y Zhao T Wu W Xiao D Liang J. Experimental and numerical studies on the compressive mechanical properties of the metallic auxetic reentrant honeycomb. Mater Des. (2019) 182:108036. 10.1016/j.matdes.2019.108036

[14] De Cock A Willems T Witvrouw E Vanrenterghem J De Clercq D. A functional foot type classification with cluster analysis based on plantar pressure distribution during jogging. Gait Posture. (2006) 23:339–47. 10.1016/j.gaitpost.2005.04.011 15990311

[15] Bousie JA Blanch P McPoil TG Vicenzino B. Hardness and posting of foot orthoses modify plantar contact area, plantar pressure, and perceived comfort when cycling. J Sci Med Sport. (2018) 21:691–6. 10.1016/j.jsams.2017.11.013 29191729

[16] Zhang X Luo Z Wang X Yang Y Niu J Fu W. Shoe cushioning effects on foot loading and comfort perception during typical basketball maneuvers. Appl Sci. (2019) 9:3893. 10.3390/app9183893

[17] Jordan C Bartlett R. Pressure distribution and perceived comfort in casual footwear. Gait Posture. (1995) 3:215–20. 10.1016/0966-6362(96)82850-5 11415701

[18] Yung-Hui L Wei-Hsien H. Effects of shoe inserts and heel height on foot pressure, impact force, and perceived comfort during walking. Appl Ergon. (2005) 36:355–62. 10.1016/j.apergo.2004.11.001 15854579

[19] Mei Q Gu Y Zheng Z Yang L Fernandez J. Foot shape, perceived comfort, and plantar pressure characteristics during long-distance running. Footwear Sci. (2017) 9:S20–S2. 10.1080/19424280.2017.1313899

[20] Shirvani H Ghasemi MH Shamsoddini A Barati K. Immediate effects of using insoles with various wedges on plantar pressure measurements and comfort level during marching. Res Square. (2021). 10.21203/rs.3.rs-258589/v1

[21] Lane TJ Landorf KB Bonanno DR Raspovic A Menz HB. Effects of shoe sole hardness on plantar pressure and comfort in older people with forefoot pain. Gait Posture. (2014) 39:247–51. 10.1016/j.gaitpost.2013.07.116 23968972

[22] Okholm Kryger K Jarratt V Mitchell S Forrester S. Can subjective comfort be used as a measure of plantar pressure in football boots? J Sports Sci. (2017) 35:953–9. 10.1080/02640414.2016.1206661 27400240

[23] Dinato RC Ribeiro AP Butugan MK Pereira IL Onodera AN Sacco IC. Biomechanical variables and perception of comfort in running shoes with different cushioning technologies. J Sci. Med. Sport. (2015) 18:93–7. 10.1016/j.jsams.2013.12.003 24444754

[24] Braunstein B Sanno M Schulze N Brüggemann G-P editors. Comfort and plantar pressure pattern during running with prefabricated insoles. In: International Society of Biomechanics in Sports (ISBS) CCSD (Centre pour la Communication Scientifique Directe) (2015).

[25] Vink P Lips D. Sensitivity of the human back and buttocks: the missing link in comfort seat design. Appl Ergon. (2017) 58:287–92. 10.1016/j.apergo.2016.07.004 27633224

[26] Fiorillo I Song Y Vink P Naddeo AJ. Designing a Shaped Seat-Pan Cushion to Improve Postural (Dis) Comfort Reducing Pressure Distribution and Increasing Contact Area at the Interface. Cambridge: Cambridge University Press (2021) p. 1113–22.

[27] Choi S Kim H Kim H. Yang WJS. A development of the self shape adjustment cushion mechanism for improving sitting comfort. Sensors. (2021) 21:7959. 10.3390/s21237959 34883963 PMC8659628

[28] Levesque L Pardoel S Lovrenovic Z Doumit M. Experimental comfort assessment of an active exoskeleton interface. In: 2017 IEEE International Symposium on Robotics and Intelligent Sensors (IRIS) (Ottawa, ON: IEEE) (2017).

[29] Yokura H Nakanishi M Niwa MJ. Using the compression properties of pillows to estimate sleeping comfort. Int J Clothing Sci Technol. (1999) 11:160–9. 10.1108/09556229910282415

[30] Goonetilleke RS. The comfort slip. In: First World Congress on Ergonomics for Global Quality and Productivity. Hong Kong (1998).

[31] Jordan C Payton C Bartlett RJCB. Perceived comfort and pressure distribution in casual footwear. Clin Biomech. (1997) 12:S5–S. 10.1016/S0268-0033(97)88312-X 11415701

[32] Witana CP Goonetilleke RS Au EYL Xiong S Lu XJE. Footbed shapes for enhanced footwear comfort. Ergonomics. (2009) 52:617–28. 10.1080/00140130802419503 19424923

[33] Lam YN Yick KL Ng SP Leung DM Yeung KL. Plantar pressure distribution and perceived comfort with elevated heel heights during standing and walking. In: 7th Textile Bioengineering and Informatics Symposium, TBIS 2014, in conjunction with the 5th Asian Protective Clothing Conference, APCC 2014. Norwalk, CT: Binary Information Press (2014).

[34] Puszczalowska-Lizis E Koziol K Omorczyk J. Perception of footwear comfort and its relationship with the foot structure among youngest-old women and men. PeerJ. (2021) 9:e12385. 10.7717/peerj.12385 34722004 PMC8532988

[35] Evans KE Nkansah M Hutchinson I. Auxetic foams: modelling negative Poisson's ratios. Acta Metallurgica et Materialia. (1994) 42:1289–94. 10.1016/0956-7151(94)90145-7

[36] Ren X Das R Tran P Ngo TD Xie YM. Auxetic metamaterials and structures: a review. Smart Mater Struct. (2018) 27:023001. 10.1088/1361-665X/aaa61c

[37] Chatrenet A Beaune B. Audebrand Jm, Torreggiani M, Piccoli GB, Morel BJ. Pedobarographic Statistical Parametric Mapping may identify specific plantar pressure patterns in patients with diabetes mellitus among different degrees of peripheral neuropathy: a pilot study. Diabet Med. (2021) 38:e14572. 10.1111/dme.14572 33783860

[38] Hamzavi B Esmaeili HJG. Effects of running-induced fatigue on plantar pressure distribution in runners with different strike types. Gait Posture. (2021) 88:132–7. 10.1016/j.gaitpost.2021.05.018 34034025

[39] Segal A Rohr E Orendurff M Shofer J O'Brien M Sangeorzan BJF. The effect of walking speed on peak plantar pressure. Foot Ankle Int. (2004) 25:926–33. 10.1177/107110070402501215 15680109

[40] Li R Zhao Y Yan S Yang L. Walking and running speed effects on plantar pressure distribution. Footwear Sci. (2017) 9:S90–S1. 10.1080/19424280.2017.1314355

[41] Chung M-J. Wang M-JJE. Gender and walking speed effects on plantar pressure distribution for adults aged 20–60 years. Ergonomics. (2012) 55:194–200. 10.1080/00140139.2011.583359 21851292

[42] Nouman M Dissaneewate T Leelasamran W Chatpun SJG. The insole materials influence the plantar pressure distributions in diabetic foot with neuropathy during different walking activities. Gait Posture. (2019) 74:154–61. 10.1016/j.gaitpost.2019.08.023 31525653

[43] Critchley R Hazael R Bhatti K Wood D Peare A Johnson S. Blast mitigation using polymeric 3D printed auxetic re-entrant honeycomb structures: A preliminary study. J Prot Struct. (2022) 13:469–86. 10.1177/20414196211052062

[44] Moroney C. The Application of Auxetic Structures for Rugby Shoulder Padding. Manchester: Manchester Metropolitan University (2021).

[45] Zwettler GA Trixner M Schartmüller C Bauernfeind S Stockinger T Praschl C. Towards an automated process for adaptive modelling of orthoses and shoe insoles in additive manufacturing (2021).

[46] Wallbanks M Khan MF Bodaghi M Triantaphyllou A Serjouei AJ. On the design workflow of auxetic metamaterials for structural applications. Smart Mat Struct. (2021) 31:023002. 10.1088/1361-665X/ac3f78

[47] Tawk C Mutlu R Alici G. A 3D printed modular soft gripper integrated with metamaterials for conformal grasping. Front Robot AI. (2022) 8:799230. 10.3389/frobt.2021.799230 35071336 PMC8782332

[48] Hurst B Branthwaite H Greenhalgh A Chockalingam N. Medical-grade footwear: the impact of fit and comfort. J Foot Ankle Res. (2017) 10:1–7. 10.1186/s13047-016-0184-z 28070223 PMC5217416

[49] Franciosa P Gerbino S Lanzotti A Silvestri L. Improving comfort of shoe sole through experiments based on CAD-FEM modeling. Med Engineer Phys. (2013) 35:36–46. 10.1016/j.medengphy.2012.03.007 22475566

[50] Yamamoto T Hoshino Y Kanzaki N Nukuto K Yamashita T Ibaraki K. et al. Plantar pressure sensors indicate women to have a significantly higher peak pressure on the hallux, toes, forefoot, and medial of the foot compared to men. J Foot Ankle Res. (2020) 13:1–7. 10.1186/s13047-020-00410-2 32611444 PMC7329404

[51] Nix SE Vicenzino BT Collins NJ. Smith MD Gait parameters associated with hallux valgus: a systematic review. J Foot Ankle Res. (2013) 6:1–12. 10.1186/1757-1146-6-9 23497584 PMC3602054

[52] Martínez-Nova A Sánchez-Rodríguez R Pérez-Soriano P Llana-Belloch S Leal-Muro A Pedrera-Zamorano JD. et al. Plantar pressures determinants in mild Hallux Valgus. Gait Posture. (2010) 32:425–7. 10.1016/j.gaitpost.2010.06.015 20643550

[53] Koenraadt KL Stolwijk NM van den Wildenberg D Duysens J Keijsers NL. Effect of a metatarsal pad on the forefoot during gait. J Am Podiatr Med Assoc. (2012) 102:18–24. 10.7547/1020018 22232317

[54] Hackney JM Hunt GC Lerche FF Voi P. Smith JW. An external shoe modification for reducing metatarsal head pressure in people with metatarsalgia. J Prosthet Orthot. (2010) 22:37–42. 10.1097/JPO.0b013e3181ccc1f1

[55] Leung MS-h Yick K-l Sun Y Chow L Ng S-p. 3D printed auxetic heel pads for patients with diabetic mellitus. Comp Biol Med. (2022) 146:105582. 10.1016/j.compbiomed.2022.105582 35588678

[56] Singh S Yoong M. Kaur AJ. Offloading techniques for diabetic foot. J Diab Metab Disorder Cont. (2017) 4:84–8. 10.15406/jdmdc.2017.04.00112

[57] James K Orkaby AR. Schwartz AW. Foot examination for older adults. Am J Med. (2021) 134:30–5. 10.1016/j.amjmed.2020.07.010 32805226 PMC9614715

[58] Gerych D Tvrznik A Prokesova E Nemeckova Z. Jelen KJ. Analysis of peak pressure, maximal force, and contact area changes during walking and running with conventional and shock-absorbing insoles in the combat boots of the Czech army. J Mech Med Biol. (2013) 13:1350042. 10.1142/S0219519413500425

